# Higher-weight social identity as a risk and protective factor in the negative health consequences of weight stigma: a systematic review

**DOI:** 10.1038/s41366-025-01755-z

**Published:** 2025-04-16

**Authors:** Alice Hudson, Luisa Batalha, Joseph Ciarrochi

**Affiliations:** 1 School of Behavioural and Health Sciences, Australian Catholic University, Strathfield, NSW Australia; 2 School of Beahvioural and Health Sciences, Australian Catholic University, North Sydney, NSW Australia

**Keywords:** Risk factors, Signs and symptoms

## Abstract

**Background:**

Weight stigma causes significant physical and psychological harm to its targets.

**Objective:**

This review aims to determine when identifying as a member of the higher-weight group exacerbates versus mitigates the adverse effects of weight stigma.

**Methods:**

Searches were conducted on 10 January, 2025, using PsycInfo, Medline, Scopus, Web of Science, Embase, and CINAHL. Evidence was synthesised in terms of exacerbating versus protective effects of higher-weight social identity (as moderator/mediator) in the relationship between weight stigma and 18 distinct health outcomes. This review is registered on PROSPERO (ID: CRD42023415639).

**Results:**

Fourteen studies met the inclusion criteria. Studies employing weight status measures to assess higher-weight social identity identified actual and self-perceived higher-weight as risk factors for anticipated rejection, dietary control challenges, increased physiological stress and greater functional disability following stigmatisation. Conversely, studies measuring individual connection with the higher-weight group revealed that stronger identification had protective effects on self-esteem and distress, but only for specific individuals (e.g., those with low internalised weight bias).

**Limitations:**

Grey literature and unpublished studies were not reviewed.

**Conclusions:**

Initial evidence suggests that higher-weight social identity functions as both risk and protective factor in the relationship between weight stigma and well-being.

**Implications:**

Future research should explore the emotional and evaluative components of higher-weight social identity to enhance understanding of how and when group membership influences the adverse effects of weight stigma. This knowledge can inform targeted interventions designed to improve the well-being of higher-weight individuals.

People with obesity are frequent targets of weight stigma (i.e., social devaluation due to body weight)^1^ across various settings including education, employment, health care, the media, and within the family home [1]. Weight stigma is manifested through negative stereotypes (e.g., lazy, incompetent, ill-disciplined) which may result in prejudice (e.g., negative attitudes) and discrimination toward the target [2]. The psychological and physical consequences of weight stigma include depression, anxiety, low self-esteem, and weight gain [3]. Interventions targeted at reducing bias against higher-weight people have had limited success to date [4]. Research has, therefore, shifted toward understanding psychosocial factors that can mitigate the negative impact of weight stigma on the targets. Two lines of research—*weight-based social identity threat* (WBSIT) [5], and *the social cure* [6, 7]—have emerged that identify weight-based social identity as a major factor in the relationship between stigma and well-being. As depicted in Fig. 1, the WBSIT model views higher-weight social identity—the part of the self-concept that comes from identifying as a member of the higher-weight social group (i.e., the ingroup) [8]—as a risk factor, while the social cure framework suggests it can be protective under specific circumstances.

**Fig. 1:**
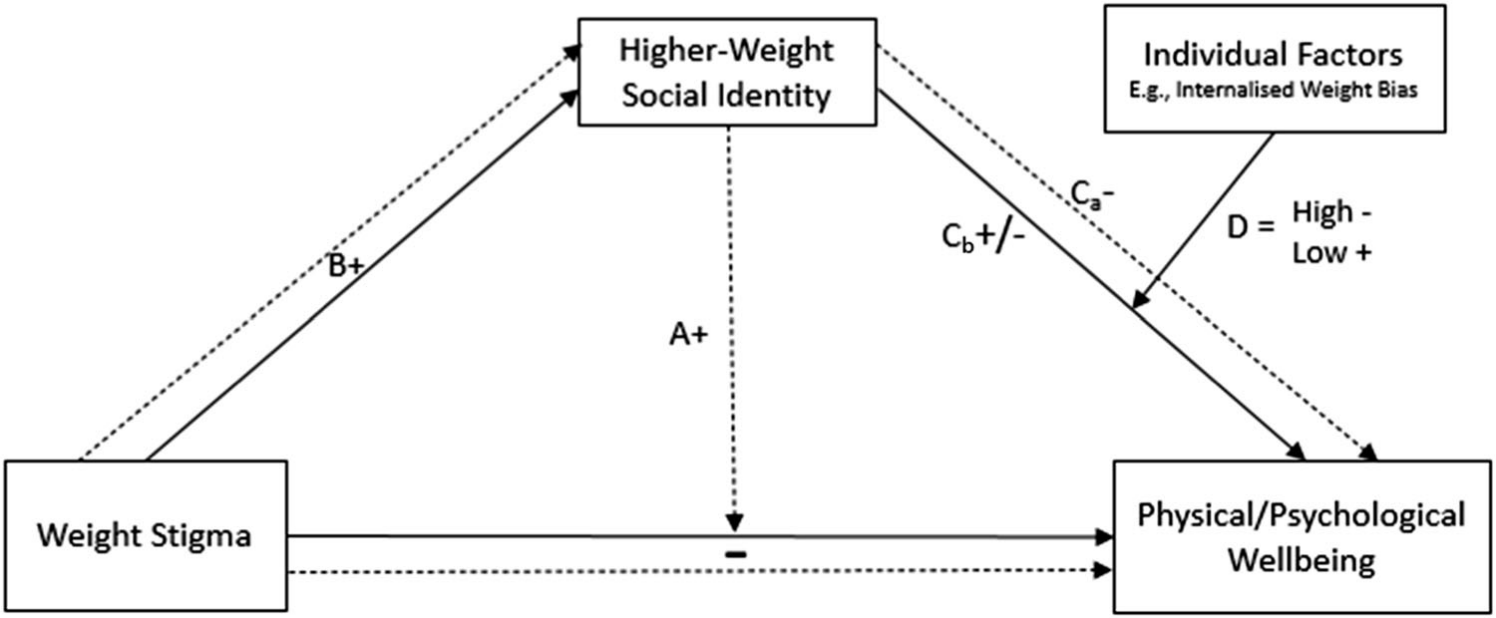
Overarching model summarising the hypothesised relationships between weight stigma, higher-weight social identity, individual factors, and health outcomes proposed by the weight-based social identity threat model and the social cure model. Note. WBSIT model denoted by dotted pathways: Higher-weight social identity is hypothesised to moderate the relationship between weight stigma and health outcomes (link A). Specifically, higher-weight social identity is predicted to exacerbate the negative effects of weight stigma on health. Higher-weight social identity is expected to mediate the relationship between weight stigma (links B and C_a_, where higher-weight social identity is negatively related to well-being). Social Cure model denoted by solid pathways: Higher-weight social identity as a detrimental mediator of the relationship between weight stigma and health outcomes (links B and C_b_−) or as a protective factor (links B and C_b_+), depending on certain circumstances (e.g., the perceived illegitimacy of stigma) and individual factors (e.g., low levels of internalised weight bias; see link D).

Identifying what constitutes risk versus protective factors is crucial as such knowledge can help inform interventions and policies aimed at reducing the adverse impacts of weight stigma on its targets and is, therefore, the general aim of this review.

## Weight-based social identity threat model, weight stigma, and health outcomes

The WBSIT model examines the adverse effects of a higher-weight social identity. As depicted by link A in Fig. 1, this model proposes that when individuals categorise themselves as higher-weight or believe others regard them as member of this social category, weight stigma increases the vulnerability to experiencing weight-based social identity threat due to fear of the association of weight stigma with the ingroup [8]. This in turn activates stress processes that can be associated with self-control resources required for regulating healthy behaviours [9]. These effects, when experienced persistently, further undermine mental and physical health and, ironically, contribute to weight gain and increase the likelihood of ongoing stigmatisation [5]. Research applying this model has also examined whether the negative impact of weight stigma on well-being is mediated by higher-weight social identification (see links B and C_a_, Fig. 1) [10].

Numerous studies support the WBSIT model. For instance, research assessing higher-weight social identity using objective weight measures like body mass index (BMI; i.e., assigned group membership), demonstrates that individuals with higher body weight experience more adverse psychological (e.g., stress emotions) [11] and physical (e.g., greater cortisol reactivity) [12] outcomes following exposure to weight stigmatisation, compared to those with lower body-weight. Other researchers suggest that self-categorisation (i.e., psychological group membership) as higher-weight is a necessary condition to elicit WBSIT and, consequently, have operationalised higher-weight social identity by measuring individuals’ self-perceived weight status in addition to BMI. For instance, Hunger et al. found that among higher-weight women and those who perceived themselves as higher-weight, exposure to weight stigma led to significantly greater anticipation of being socially rejected because of their weight compared to their average-weight counterparts [13].

Although the WBSIT perspective provides useful insights into the mechanisms connecting weight stigma and adverse health outcomes among higher-weight individuals, this approach is limited by its conceptualisation of higher-weight social identity as an individual’s mere *awareness* of belonging to the higher-weight group. Evidence suggests that the emotional and evaluative significance of group membership to an individual is also likely to have important implications for health outcomes [14]. This evidence is consistent with influential definitions of social identity, which includes both *knowing* one belongs to a group and *feeling attached* to it through emotions and/or values [15].

In addition to these methodological and conceptual issues, WBSIT research may also be limited by the assumption that higher-weight individuals universally internalise weight stigma [16, 17] and do not display ingroup favouritism (see Crandall [18]), a well-documented tendency among ingroup members [19]. However, recent studies show that many individuals actively resist weight stigma (e.g., “Big is Beautiful” reframing strategies), and that ingroup identification is an important predictor of this resistance [20]. To address these limitations, some researchers have turned to the social cure approach to examine weight stigma and health outcomes.

## The social cure approach, weight stigma, and health outcomes

Informed by social identity theory [21], and self-categorisation theory [22], the social cure approach [6, 7] proposes that although some social identities (particularly low status social identities) may be a potential source of distress (i.e., a social curse—see Cruwys and Gunaseelan [23], Kyprianides et al. [24]), they may also act as a buffer against adverse consequences of stigma (i.e., a social cure; see links B and C_b_, Fig. 1). In contrast to the WBSIT model, which limits its focus on group membership awareness, this approach considers the broader definition of social identity (see Postmes et al. [14]) that includes an individual’s *evaluative* and *emotional* connection to the ingroup (which may vary in strength and be positive/negative/neutral). Drawing from the rejection-identification model (RIM) [25], the social cure approach proposes that stigmatised individuals, who perceive group boundaries as impermeable and discrimination toward the ingroup as unfair, are likely to respond to stigma by identifying with ingroup members to reduce feelings of rejection, which in turn, protects well-being by increasing perceived social support [26]. Members may also pursue collective social strategies that provide an additional basis for social support and promote hope and optimism [27].

In the context of weight, the pervasive discrimination and perceived impermeability of group boundaries, due to the challenge of achieving long-term weight loss [28], are likely to move stigmatised higher-weight people closer to their ingroup [20]. In this vein, the RIM asserts that higher-weight social identification plays a mediating role in the relationship between weight stigma and well-being with a positive effect on well-being (see links B and C_b_+, Fig. 1). However, the highly negative content of weight stigma is commonly endorsed by group members [28]. For those members, the social cure approach predicts that ingroup identification may lead to a social curse rather than to a social cure (see links B and C_b_−, Fig. 1).

Given these complexities, and the current lack of research examining protective factors [2], researchers have recently sought to clarify when and for whom identification with the higher-weight group is likely to buffer against the harmful effects of weight stigma (e.g., Curll and Brown [29]; Magallares et al. [30]). Internalised weight bias (IWB), which is strongly associated with negative mental health outcomes [31] and research shows, for example, that individuals with high IWB are more likely to legitimise weight stigma [32]. Curll and Brown [29] tested the role of IWB within the social cure model and predicted that the social cure effect would depend on individual differences in IWB. They operationalised higher-weight social identification as the degree to which being higher-weight was central to a participant’s identity (identity centrality). Results indicated that stronger higher-weight social identification was associated with lower psychological distress (i.e., improved well-being; see links B and D, Fig. 1), but only among individuals with low IWB. This study is important as it provides a more nuanced understanding of the factors influencing the relationship between weight stigma and the mental and physical health of higher-weight individuals by considering both group (i.e., social identity) and individual-level (i.e., IWB) factors.

## Rationale and objectives

Thus far, interventions on weight stigma have mostly focused on reducing stigmatisation (i.e., focus on the stigmatisers), and have generally been unsuccessful [4]. Because weight stigma produces highly detrimental health effects on targets, it is important to investigate risk and resilience factors that may exacerbate or prevent/reduce the harmful impact of stigma. To date, no synthesis of this literature exists.

Taken together, the literature reviewed above suggests that social identity can be both a cure and curse depending on context, and that individual factors can play a role in determining the outcome (e.g., IWB). Therefore, this review will not test the validity of the WBSIT or the social cure models. Instead, it aims to test the two models together and review studies that have explored how higher-weight social identity moderates and/or mediates the relationship between weight stigma and psychological and physical well-being (i.e., WBSIT model). In addition, it also aims to investigate whether the mediating effect of higher-weight social identity is moderated by individual factors (such as perceived il/legitimacy of stigma, or IWB; i.e., the social cure model). The ultimate goal is to understand whether and when higher-weight social identity exacerbates versus reduce weight stigma harm. As such, this review will provide a synthesis of the state-of-the art in this field, contribute with suggestions for future research, and offer evidence-based recommendations for targeted interventions and policies aimed at improving health outcomes for higher-weight individuals.

## Methods

The preferred reporting items for systematic review and meta-analyses (PRISMA) statement [33] were followed for this review. Methods were established prior to data extraction and pre-registered in PROSPERO (ID: CRD42023415639).

## Search strategy

Systematic searches were performed on January 10, 2025, using PsycINFO, CINAHL, Web of Science Core Collection, Medline Complete, Scopus, and Embase databases with no date limitations on studies. Search results were imported into Covidence via Endnote for screening. A combination of search terms using Boolean phrasing (see Table 1 in Supplementary Materials) were used to identify papers relevant to weight stigma, social identity, and health outcomes. Additional studies were identified via a search of reference lists of included papers.

## Eligibility criteria

To be eligible, studies had to be peered reviewed and meet the following criteria: there had to be (1) a measure of experienced or perceived weight stigma as an independent variable, (2) a measure of higher-weight social identity/identification (as a mediator or a moderator variable), and (3) a physical or psychological health outcome measure. Studies were excluded if they: (1) were reports, reviews, book chapters, abstracts, dissertations, were not peer reviewed, or provided insufficient data, (2) were unpublished studies, (3) included the wrong independent variable, mediator or moderator, or outcome measure, and (4) were not written in English, due to interpretation limitations.

## Screening and data extraction

Studies were independently screened by two reviewers (AH and LB) at both the title and abstract, and full-text screening stages using Covidence. Any disagreements were resolved through discussion. The data from eligible studies were extracted by AH and included (if reported): author name(s), country, year of publication, sample characteristics (sample size, source, percentage of females), age, BMI (mean and standard deviation), study design (i.e., cross-sectional, longitudinal, or experimental), and measure(s) of the independent variable, the mediator/moderator, and the physical and/or mental health outcome variables. Inferential statistics and standardised effects sizes were extracted for all statistical analyses. Guidelines published by Cohen [34] were used for assessing effect sizes.

## Risk of bias assessment

A risk of bias assessment was conducted by AH and LB using the Centre for Evidence-Based Management (CEBMa) checklist for surveys, irrespective of study design. This approach was adopted in a recent systematic review for weight stigma effects (see Hill et al. [35]) to allow comparison across studies with a single tool (all eleven criteria are displayed in Table 2 in Supplementary Materials). Each criterion was assessed as “yes,” “no,” or “can’t tell.” Overall quality rating (i.e., good, fair, or poor) was assessed independently by AH and LB based on the critical appraisal of the risk of potential for selection, information bias, measurement, or confounding. Any conflicts were discussed and resolved between AH and LB.

## Results

### Study search

Following a search of six databases, the studies were entered into Covidence, which identified duplicates and automatically removed them before they were screened by title and abstract. Of the initial 1855 studies 57 remained to be screened against the full-text eligibility criteria, which resulted in 14 studies left for inclusion in the review (see Fig. 1 in Supplementary Materials and Appendix A in Supplementary Materials).

### Higher-weight social identity as a risk factor

#### Study characteristics

Table 1 shows a summary of the characteristics for each of the 10 included studies examining higher-weight social identity as a risk factor (*N* = 5955). That is, studies that evaluated link A, as well as links B and C_a_ in Fig. 1. All studies were published between 2011 and 2025, with nine studies conducted in the United States of America, and one in China. Four studies recruited only women. Study designs were mixed, with seven using between-subjects, two using cross-sectional single sample (correlational), and one using a longitudinal design.

**Table 1:** Characteristics of studies of higher-weight social identity as a risk factor.

Study (year, country)	Design	Sample characteristics					Weight stigma measure	HWSI measure	Outcome(s) measure	Conclusion
		*N*	Source	Age *M* (SD)	Female (%)	BMI *M* (SD)				
Blodorn et al. [47] USA	Experimental 2 between-groups EC: Belief that speech on dating qualities could be seen by evaluator CC: Belief that speech could not be seen (weight unseen)	160	Student	20.88 (2.95)	52	25.93 (5.21)	Experimentally manipulated AWS in a speech on dating qualities scenario	BMI	Rejection expectations: (Likert responses to 8 author-created questions regarding rejection expectations) Executive function: Performance on Stroop colour naming task to measure cognitive depletion State self-esteem: (SSES) Self-conscious emotions: (4 author-created items assessing negative self-conscious emotions) Stress emotions: (Likert responses to questions regarding level of stress, after speech)	BMI did not moderate the relationship between weight stigma and outcomes assessed, i.e., rejection expectations, executive functioning, state self-esteem, self-conscious emotions, stress emotions. BMI only moderated the relationship between weight stigma and outcomes assessed for women.
Brochu and Dovidio [49] USA	Experimental 2 between-groups Weight stigma manipulation: EC: Participants asked to read vignette describing a fictious study containing weight stigma material CC: neutral vignette	176	Community	33.66 (12.45)	52.27	26.21 (6.62)	Experimentally manipulated weight stigma in a food choice scenario	BMI	Total calories ordered from dinner menu	BMI moderated the relationship between weight stigma and number of calories ordered. The weight stigma manipulation significantly increased the number of calories ordered by higher-weight participants (BMI > 27.89) but did not influence food selection food selection of participants with a BMI < 27.89.
Himmelstein et al. [56]	Experimental 2 between-groups Weight manipulation: EC: Shopping activity with weight stigma scenarios CC: Control (did not participate in the shopping activity)	110	Student	19.77 (4.76)	100	19.30 (1.55)	Experimentally manipulated weight stigma in a clothes shopping scenario	Self-perceived bodyweight	Saliva cortisol level at baseline and 30 min post-manipulation (to see the stress-responsive hypothalamic-pituitary- adrenal-axis)	Perceptions of body weight moderated the effect of weight stigma on cortisol reactivity. For individuals with higher self-perceived body weight there was a significant, positive effect of weight stigma on cortisol reactivity. Namely, those in the control condition showed a diurnal decline in cortisol levels from baseline to post-manipulation, whereas those in the weight stigma condition maintained their pre-stressor cortisol levels. For average self- perceived body weight there was no effect of weight stigma on cortisol reactivity.
Hunger et al. [13] USA	Experimental 2 between-groups Weight stigma manipulated: EC: Participants interacted with same sex anti- fat peer CC: Participants interacted with unbiased partner	146	Student	19.95 (2.18)	100	30.61 (4.34)	Experimentally manipulated weight stigma in a social interaction scenario	BMI Self-perceived bodyweight	Anticipated rejection: (Likert responses to 8 author-created questions regarding rejection expectations) Stress: (Likert response to one question “I found the interaction stressful”) Cognitive performance: Responses on a word- finding game State self-esteem: (SSES) Self-conscious emotions: (5 items)	1. Both BMI and self- perceived weight moderated the effects of weight stigma on anticipated rejection. Higher BMI and self- perceived weight were significantly associated with anticipated rejection in the weight stigma condition (compared to control). In contrast, this association did not differ significantly between conditions among lower-weight women. 2. Neither BMI nor self-perceived weight significantly moderated the relationship between weight stigma and: a) Cognitive performance b) state self-esteem, c) self-conscious emotions d) post-interaction rumination anxiety
Lee et al. [37] USA	Cross-sectional single sample, correlational	1327	Community	47.70 (17.20)	50.4	28.0 (7.40)	2-item composite measure of daily AWS and EWS	BMI Self-perceived bodyweight	Disordered eating: (EDE-QS) Alcohol use: Alcohol Use Disorders Identification Test-C	BMI and perceived weight status significantly moderated the association between weight stigma and disordered eating and alcohol use. Unexpectedly, participants with lower BMIs and lower body-weight perception showed significantly greater disordered eating and alcohol use.
Major et al. [11] USA	Experimental 2 between- groups Weight stigma manipulated: EC: Believed speech about dating qualities could be seen (weight seen) CC: Believed speech could not be seen (weight unseen)	99	Students	18.83 (1.33)	100	27.40 (5.64)	Experimentally manipulated AWS in a speech on dating qualities scenario.	BMI	Executive control: Performance on Stroop colour naming task to measure cognitive depletion Subjective stress: Likert responses to items regarding feelings of stress during speech	Compared to lower- weight women (1.25 SD below mean BMI), higher-weight women (1.25 SD above mean BMI) in the EC (versus CC) reported significantly greater subjective stress, and significantly worse executive control.
Major et al. [50] USA	Experimental 2 between- groups Weight stigma manipulated: EC: Read news article about weight stigma in job market CC: Read neutral article	93	Student	19.15 (NR)	100	24.28 (4.71)	Experimentally manipulated AWS in a news article reading activity	Self-perceived bodyweight	Self-regulation depletion: consumption of calorie dense food Self-efficacy for dietary control: adapted from existing self-efficacy scale by Clark et al. [57] Weight stigma concerns: Likert responses to questions regarding weight stigma concerns	1. Exposure to weight stigmatising news articles led participants who perceived themselves as overweight (but not those who did not perceive themselves as overweight) to: a) consume more calories a) feel less capable of controlling their food intake, compared to those not exposed to stigmatising news articles. 2. The significant, positive effect of weight stigma on weight stigma concerns was not significantly moderated by self-perceived weight.
McCleary -Gaddy et al. [53] USA	Experimental 2 between- groups Weight stigma manipulated: EC: Asked to make a supposedly videotaped speech (weight seen) about what makes them a good candidate for a job at a company with weight discriminatory policies. CC: Made a supposedly audiotaped (weight unseen) speech for a Company.	170	Community	35.01 (9.83)	60.59	27.36 (6.39)	Experimentally manipulated AWS in a job interview scenario. Participants exposed to a laboratory stressor, modelled after the Trier Social Stress Test.	BMI Self-perceived bodyweight.	Cortisol reactivity: Analysis of cortisol samples (baseline, peak, and two recovery samples). Note: Increases in cortisol from baseline to post-test manipulation (rather than diurnal cortisol decline; see Himmelstein et al. [56]) were measured	Both BMI and self- perceived weight moderated the relationship between weight stigma and cortisol reactivity. For lean participants, exposure to weight stigma (compared to control) induced increased cortisol levels. For overweight (actual and self-perceived) participants, weight stigma (versus control) induced blunted cortisol responses. Note: This response is consistent with research indicating that people who have experienced chronic stress respond to acute stressors with a blunted cortisol response.
Schafer and Ferraro [10] USA	Longitudinal (Follow up: 10 years)	1856	Community	46.56 (12.69)	51.62	26.69 (5.30)	PWD–author-created questions about appraisals of weight-based discriminatory experiences	Self-perceived weight status	Functional disability change: (responses to 9 questions regarding activity limits)	Weight perceptions fully mediated the relationship between perceived weight discrimination and increases in functional disability.
Wang et al. [38] China	Cross-sectional	1818	Students	16.5 (0.98)	46	21.2 (3.52)	POTS	BMI	Eating behaviours: TFEQ-R18	The relationship between weight stigma and eating behaviours differed between non-overweight participants and overweight or obese participants. The experience of weight stigma is significantly associated with greater levels of uncontrolled eating, emotional eating, and cognitive restraint among non- overweight adolescents. The experience of weight stigma was significantly associated with greater levels of uncontrolled eating and emotional eating, but not cognitive restraint among overweight or obese.

*EC* experimental condition, *CC* control condition, *AWS* anticipated weight stigma, *EWS* experienced weight stigma, *IWS* internalised weight stigma. Measure Acronyms. Predictor: *POTS* perception of teasing scale, *PWD* perceived weight discrimination, *PWS* perceived weight stigma. Outcome: *TFEQ-R18* three-factor eating questionnaire, *EDE-QS* Eating disorder examination questionnaire, *SSES* state self-esteem scale.

##### Weight stigma measures

Most studies (*n* = 7) utilised different weight stigma manipulation paradigms to experimentally induce weight stigma (see Table 3 in Supplementary Materials) and examine its impact on individuals’ well-being. One study used an author-created questionnaire to measure perceived weight stigma, one used the original version of the Perception of Teasing Scale [36], and one used a 2-item composite validated measure of daily anticipated and experienced weight stigma [37].

##### Higher-weight social identity measures

Four studies measured BMI as an indicator of higher-weight social identity, and three measured self-perceived weight. Only three studies measured both assigned and psychological membership (i.e., BMI and self-perceived weight, respectively).

##### Outcome measures

Twelve different psychological outcomes and two physical outcomes were examined. The most common psychological outcome was executive functioning (*n* = 3). Each of the following were examined in two studies: self-regulation, rejection expectations, self-conscious emotions, state self-esteem, stress emotions, and disordered eating. Each of the following were examined in one study only: self-efficacy for dietary control, weight stigma concerns, anxiety, post-interaction rumination, and alcohol use. Cortisol reactivity was examined in two studies and functional disability (i.e., change in mobility over a 10-year period) in one.

### Synthesis of results

As displayed in Tables 2–4, there were 31 unique findings resulting from the nine studies that examined higher-weight social identity as a moderator (i.e., tested link A in Fig. 1). One study (see Table 5) examined the mediating role of higher-weight social identity (i.e., tested links B and C_a_ in Fig. 1). All moderating/mediating effects were in line with the studies’ hypothesised direction, with two exceptions in Lee et al.’s [37] study (see below), and 50% were statistically significant.

**Table 2:** Results for the relationship between weight stigma and outcomes related to consumption behaviours (*n* = 4) moderated by higher-weight social identity (For an explanation of statistics presented in this Table, refer to Conclusions in Table 1).

Outcomes of weight stigma	Study	Moderation			Summary	*k*^b^	*k* significant %
		*p* (Δ*F/F*)	Δ*R*^2a^	Simple slopes *β* (95% CI)			
Self-regulation-increased calorie consumption (*n* = 2)						2	100%
	Brochu and Dovidio [49] • BMI as a moderator	*b* = 44.49**	NR [*R*^2^ = 0.41**]	BMI > 27.89: *βJN* = 222.67* (NR)	Significant moderating effect of BMI: high levels of BMI: significant, positive effect of weight stigma on calorie consumption Low levels of BMI: no effect of weight stigma on calorie consumption. Effect size of interaction not reported		
	Major et al. [50] • Self-perceived weight as a moderator	*β* = 0.33**	NR	+1 SD above mean perceived weight: 0.37** (NR) −1 SD below mean perceived weight: −0.12 (NR)	Medium, significant moderating effect of self-perceived weight. High levels of self-perceived weight: significant, positive effect of weight stigma on calorie consumption. Low levels of self-perceived weight: no effect of weight stigma on calorie consumption.		
Self-efficacy for dietary control (*n* = 1)						1	100%
	Major et al. [50] • Self-perceived weight as a moderator	*β* = −0.44**	NR	+1 SD above mean perceived weight: −0.32** (NR) −1 SD below the mean perceived weight: 0.32** (NR)	Medium, significant moderating effects of self-perceived weight. High levels of self-perceived weight: significant, negative effect of weight stigma on self-efficacy for dietary control. Low levels of self-perceived weight: stigma on self-efficacy for dietary control.		
Disordered eating (*n* = 2)						3	100%
	Lee et al. [37] Regression moderation • BMI as moderator • Self-perceived weight status as moderator	*b* = 0.01*** *b* = −0.04***	NR NR	BMI < 32.09: *β*JN = significant BMI > 55.51: *β*JN = non-significant NR	BMI and perceived weight status significantly moderated the association between weight stigma and disordered eating. Lower levels of BMI and lower body-weight perception: significant, positive effect of weight stigma on disordered eating. Lower levels of BMI and lower body-weight perception: significant, positive effect of weight stigma on disordered eating. Higher levels of BMI and higher body-weight perception: no effect of weight stigma on disordered eating Effect size of interaction effect not reported.		Note: Unexpectedly, positive relationships between weight stigma and disordered eating were weaker with stronger higher-weight social identification.
	Wang et al. [38] • BMI as moderator	*NR*	*NR*	NR	Significant moderating effects of BMI Non-overweight: weight stigma experiences associated with significantly greater levels of uncontrolled eating (*β* = 1.61***, SE = 0.02); emotional eating (*β* = 1.99***, SE = 0.01); and cognitive restraint (*β* = 2.04***, SE = 0.01). Overweight or obese: weight stigma experiences associated with significantly greater levels of uncontrolled eating (*β* = 0.29***, SE = 0.03); emotional eating (*β* = 0.27*** SE = 0.01), but not with cognitive restraint.		
Alcohol use (*n* = 1)						2	100%
	Lee et al. [37] – Regression moderation • BMI as moderator • Self-perceived weight status as moderator	*b* = −0.03** *b* = −0.18**	NR NR	BMI < 32.09: *β*JN = significant BMI > 55.15: *β*JN = nonsignificant NR	BMI and perceived weight status significantly moderated the association between weight stigma and alcohol use. Lower levels of BMI and lower body-weight perception: significant, positive effect of weight stigma on disordered eating. Higher levels of BMI and high body-weight perception: no effect of weight stigma on disordered eating. Effect size of interaction effect not reported.		Note: Unexpectedly, positive relationships between weight stigma and alcohol use were weaker with stronger higher-weight social identification.

All studies employed a regression moderation.

*β* standardised regression coefficient, *b* unstandardised regression coefficient, *NR* not reported.

*p < 0.05; **p < 0.01; ***p < 0.001.

^a^Δ*R*^2^ = inferential statistics for the effect size of interaction effects of higher-weight social identity and weight stigma on outcomes assessed.

^b^*k* = number of unique findings reported across the studies.

**Table 3:** Results for the relationship between weight stigma and other psychological outcomes (*n* = 4) moderated by higher-weight social identity (For an explanation of statistics presented in this Table, refer to Conclusions in Table 1).

Outcomes of weight stigma	Study	Moderation			Summary	*k*^b^	*k* significant %
		*p* (Δ*F/F*)	Δ*R*^2a^	Simple slopes *β* (95% CI)			
Rejection expectations (*n* = 2)						3	66.67%
	Blodorn et al. [47] • BMI as a moderator Hunger et al. [13] • BMI as moderator • Self-perceived weight as a moderator	1.58 5.93* 4.07*	0.02 0.04 0.02	– +1 SD: 0.44*** (NR) Mean: 0.26** (NR) −1 SD: −0.15 (NR) Very overweight: 0.40*** (NR) Slightly overweight: 0.09 (NR)	No moderating effects of BMI. Significant moderating effects of BMI on the relationship between weight stigma and rejection expectations only found for women. Small, significant moderating effects of BMI and self-perceived weight. High levels of BMI and perceived very overweight: significant, positive effect of weight stigma on rejection expectations. Low levels of BMI and perceived slightly overweight: no effect of weight stigma on rejection expectations.		
Executive functioning (*n* = 3)						4	25%
	Blodorn et al. [47] • BMI as moderator	0.74	0.01	–	No moderating effect of BMI on the relationship between weight stigma and executive functioning.		
	Hunger et al. [13] • BMI as moderator • Self-perceived weight as moderator Major et al. [11] • BMI as moderator	1.56 2.17 NR	0.01 0.01 0.05*	– – +1.25 SD: 0.32* (NR) −1.25 SD: -0.26 (NR)	No moderating effect of BMI or self-perceived weight on the relationship between weight stigma and executive functioning. Small, significant moderating effects of BMI. High levels of BMI: significant, positive effect of weight stigma on executive functioning Low levels of BMI: no effect of weight stigma on executive functioning.		
Self-conscious emotions (*n* = 2)						3	0%
	• Blodorn et al. [47] • BMI as a moderator Hunger et al. [13] • BMI as moderator • Self-perceived weight as moderator	1.30 0.27 0.00	0.02 0.00 0.01	– – –	No moderating effects of BMI. Significant moderating effects of BMI on the relationship between weight stigma and self-conscious emotions only found for women. No moderating effects of BMI or self-perceived weight on the relationship between weight stigma and self-conscious emotions.		
State self-esteem (*n* = 2)	Blodorn et al. [47] • BMI as moderator HHunger et al. [13] • BMI as moderator • Self-perceived weight as a moderator	2.20 0.27 0.00	0.02 0.00 0.01	– – –	No moderating effects of BMI. Significant moderating effects of BMI on the relationship between weight stigma and state self-esteem only found for women. No moderating effects of BMI or self-perceived weight on the relationship between weight stigma and state self-esteem	3	0%
Stress emotions (*n* = 2)						2	50%
	Blodorn et al. [47] • BMI as moderator Major et al. [11] • BMI as moderator	NR	0.00 0.05*	– +1.25 SD*:* 0.39* −1.25 SD: −0.18 (NR)	No moderating effects of BMI. Significant moderating effects of BMI on stress emotions only found for women. Small, significant moderating effects of BMI. High levels of BMI: significant, positive effect of weight stigma on stress emotions		
Weight stigma concerns (*n* = 1)						1	0%
	Major et al. [50] • Self-perceived weight as moderator	*β* = 0.12	NR	NR	No moderating effect of self-perceived weight on the relationship between weight stigma and weight stigma concerns.		
Post-interaction rumination (*n* = 1)						2	0%
	Hunger et al. [13] • BMI as moderator • Self-perceived weight as moderator	0.96 0.30	0.01 0.00	*–* *–*	No moderating effect of BMI or self-perceived weight on the relationship between weight stigma and post- interaction rumination.		
Anxiety (*n* = 1)						2	0%
	Hunger et al. [13] • BMI as moderator • Self-perceived weight as moderator	1.64 1.05	0.09 0.03	– –	No moderating effect of BMI or self-perceived weight on the relationship between weight stigma and anxiety.		

All studies employed a regression moderation.

*β* standardised regression coefficient, *b* unstandardised regression coefficient, *NR* not reported.

*p < 0.05; **p < 0.01; ***p < 0.001.

^a^Δ*R*^2^ = inferential statistics for the effect size of interaction effects of higher-weight social identity and weight stigma on outcomes assessed.

^b^*k* = number of unique findings reported across the studies.

**Table 4:** Results for the relationship between weight stigma and physical outcomes (cortisol reactivity) moderated by higher-weight social identity (For an explanation of statistics presented in this Table, refer to Conclusions in Table 1).

Moderation								
Outcomes of weight stigma	Study	*p* (Δ*F/F*)	Δ*R*^2a^	_*p*_*η*^2b^	Simple slopes *β* (95% CI)	Summary	*k*^c^	*k* significant %
Physiological stress: cortisol reactivity (*n* = 2)							3	100%
	Himmelstein et al. [56] • Self-perceived weight as moderator McCleary-Gaddy et al. [54] – regression moderation • BMI as moderator • Self-perceived weight as moderator	13.48*** 5.61** 5.71**	– 0.04** 0.12**	0.12*** – –	– 75th percentile: 3.55 (−10.94, 3.84) 50th percentile: 2.64 (−2.60, 7.89) 25th percentile: 8.84** (1.51, 16.17) 75th percentile: −3.58 (−10.83, 3.67) 50th percentile: 2.63 (−2.57, 7.81) 25th percentile: 8.83** (1.48, 16.17)	Perceptions of body weight moderated the effect of weight stigma on cortisol reactivity. Small, significant interaction effect. Higher self-perceived body weight: significant, positive effect of weight stigma on cortisol reactivity. Those in the control condition showed a diurnal decline in cortisol levels from baseline to post-manipulation, whereas those in the weight stigma condition maintained their elevated pre-stressor cortisol levels. Average self-perceived body weight: no effect of weight stigma on cortisol reactivity. Small to medium, significant moderating effect of BMI and self-perceived weight, respectively. Higher BMI and self-perceived body weight: weight stigma not associated with elevated cortisol (demonstrated blunted cortisol response to an acute stressor). Low BMI and self-perceived body weight: weight stigma associated with increased cortisol levels.		

The McCleary-Gaddy et al. [53] study employed a regression moderation. The Himmelstein et al. [56] study used ANCOVA.

*β* standardised regression coefficient, *NR* not reported.

**p < 0.01; ***p < 0.001.

^a^Δ*R*^2^ = inferential statistic for the effect size of interaction effects of higher-weight social identity and weight stigma on outcomes assessed using regression moderation in the McCleary-Gaddy et al. [53] study.

^b^*pη*^2^ = inferential statistic for the effect size of interaction effects of higher-weight social identity and weight stigma on outcomes assessed using ANCOVA in the Himmelstein et al. [56 study.

^c^*k* = number of unique findings reported across the studies.

**Table 5:** Results for the relationship between weight stigma and functional disability mediated by higher-weight social identity (For an explanation of statistics presented in this Table, refer to Conclusions in Table 1).

Outcome of weight stigma	Study	Mediation analysis	Summary	*k*^a^	*k* significant %
Functional disability (*n* = 1)				1	100
	Schafer and Ferraro [10]	Multinomial logistic regression to test the initial path (i.e., the association of PWD and self-perceived weight status). Ordinary least squares regression to test indirect effects of PWD on disability through weight perceptions.	1. PWD contributed to likelihood of feeling somewhat overweight (versus not overweight) (Relative Risk Ratio [RRR]) = 2.58* (95% CI = 1.02–6.25) and the likelihood of feeling very overweight (versus not overweight) RRR = 4.08* (95% CI = 1.35–12.34) perceived weight status. 2. Significant relationship between feeling very overweight and an increase in disability but non-significant effects for those who felt only somewhat overweight. 3. PWD was significantly associated with greater likelihood of functional disability (*F* = 4.73**). Evidence for mediating effect of HWSI: when including weight perception in the model, the effects of PWD on disability became nonsignificant (suggesting full mediation) for obese participants and were somewhat attenuated for severely obese participants, suggesting partial mediation (*b* = 0.27*, from 0.38**). However, indirect effect not reported.		

*PWD* perceived weight discrimination, *HWSI* higher-weight social identity.

*p < 0.05; **p < 0.01.

^a^*k* = number of unique findings reported in the study.

### Psychological outcomes

#### Outcomes related to consumption behaviours

As shown in Table 2, outcomes relating to calorie consumption behaviours were the most affected by weight stigma and higher-weight social identity. Specifically, compared to a control group, following exposure to weight stigma, only participants who were higher-weight, or perceived they were, consumed more calories and felt less capable of dietary control. Interestingly, weight stigma was associated with greater restrictive eating among *lower* compared to higher-weight individuals [38], and Lee et al. [37] found that the positive relationships between weight stigma and (1) disordered eating and (2) alcohol use were weaker when identification was stronger.

#### Other psychological outcomes

Table 3 shows results for other psychological outcomes. Two out of three findings from studies that assessed rejection expectations indicated that higher-weight social identity exacerbated the negative impact of weight stigma. Results were mixed for stress emotions and executive functioning. Higher-weight social identity did not significantly moderate the relationships between weight stigma and self-conscious emotions, state self-esteem, weight stigma concerns, post-interaction rumination, and anxiety.

### Physical outcomes

As shown in Tables 4 and 5, there were four unique findings across all studies for physical outcomes. Across the two studies that examined cortisol reactivity, higher-weight social identity was found to significantly increase the harmful effects of weight stigma on this outcome (see Table 4). Lastly, Schafer and Ferraro [10] found that perceived weight discrimination was positively associated with self-perceived higher-weight, which significantly mediated the harmful effect of perceived weight stigma on functional disability (see Table 5).

### Higher-weight social identity as a protective factor

#### Study characteristics

Table 6 summarises the characteristics for each of the four studies that examined higher-weight social identity as a protective factor (*N* = 3017). That is, studies that tested links B, C_b_, and D in Fig. 1. One study was conducted in Spain, one in Australia, and two in the USA. All studies used a cross- sectional, correlational design.

**Table 6:** Characteristics of higher-weight social identity as a protective factor studies.

Study (year, country)	Design	*N*	Sample characteristics			BMI *M* (SD)	Weight stigma measure	HWSI measure	Outcome(s) measure	Conclusion
			Source	Age *M* (SD)	Female (%)					
Curll and Brown [29]	Cross- sectional—single sample	458	Community	35.5 (11.62)	82.3	35.72 (7.45)	EDS to measure PWS	GIS-C	Psychological distress: (PDS, K10)	Positive indirect effect of PWS on psychological distress through HWSI (links A and C_b_, Fig. 1). However, this indirect effect was moderated by IWB (link D, Fig. 1) such that: 1. Among participants with low IWB, HWSI was associated with lower psychological distress; but for participants with high IWB, HWSI was associated with greater psychological distress. 2. PWS was associated with lower psychological distress via HWSI (negative indirect effect) at low levels of IWB but this indirect effect increased and became positive with higher levels of IWB (see link D, Fig. 1)
Magallares et al. [30]	Cross- sectional—single sample	95	Community	39.54	66.3	42.61 (9.76)	OP	CSES	Self-esteem: (RSES)	Path analysis showed a significant negative relationship between weight stigma and self-esteem but the relationship between higher- weight group identification and self- esteem was positive and significant (see link C_b_, Fig. 1)

Wellman et al. [45]	Cross- sectional—single sample	739	Community	53.45 (16.61)	61.7	NR	PDS	FIS	Self-Esteem: (RSES) Satisfaction with life: (SWLS) General Health: SF-12	Negative, significant indirect effect of PWS on self-esteem and general health through HWSI. That is, higher PWS was negatively related to self-esteem and general health, and the indirect pathways from weight stigma to HWSI to self-esteem and general health were also negative (see link C_b_, Fig. 1). HWSI was found not to be a significant mediator of the relationship between weight stigma and general life satisfaction.
Azaira et al. [44]	Cross- sectional—single sample	1725	Community	55.83 (16.02)	71.0	NR	PDS	FIS	Perceived Stress: (PSS) Eating behaviours: (TFEQ-R18)	Negative, significant indirect effect of PWS on perceived stress and disordered eating (uncontrolled eating and emotional eating) through HWSI. That is, higher PWS was negatively related to perceived stress, uncontrolled eating and emotional eating, and the indirect pathways from weight stigma to HWSI perceived stress, uncontrolled eating and emotional eating were also negative (see link C_b_, Fig. 1). HWSI was found not to be a significant mediator of the relationship between weight stigma and general life satisfaction.

*HWSI* higher-weight social identification, *PWS* perceived weight stigma. Measure acronyms. Predictor: *OP* Obesity-Related Problems Scale, *EDS* Everyday Discrimination Scale, *PDS* Perceived discrimination scale. Mediator: *CSES* Group Identification Scale, *GIS-C* 5-factor group identification scale, adapted to apply to an overweight identity, centrality subscale, *FIS* Fat identification scale. Moderator: *IWB* internalised weight bias: Outcome: *PSS* Perceived Stress Scale, *RSES* Rosenberg self-esteem scale, *PDS (K10)* Psychological distress scale, *SWLS* Satisfaction with life scale, *RSE* Rosenberg Self-Esteem Scale, *SF-12* Short form Health Survey, *TFEQ-R18* three-factor eating questionnaire.

##### Weight stigma measures

The studies used different measures of weight stigma. One used the Everyday Discrimination Scale [39], one used the Obesity Related Problems Scale [40], and two used the Perceived Discrimination Scale [41].

##### Higher-weight social identity measures

Different measures were also used to assess higher-weight social identification. These included the centrality subscale of the 5-factor Group Identification Scale [42], the centrality subscale of the Group Identification Scale [43], and the Fat-Group Identification Scale [41].

##### Outcome measures

Across the four studies, self-esteem, satisfaction with life, stress, psychological distress, disordered eating, and general health were assessed as outcomes.

### Synthesis of results

As shown in Table 7, there were seven unique findings across the four studies. Four findings were in line with the authors’ predictions [29, 30, 44] and two were in the opposite direction (see below; Wellman et al. [45]). Six of the seven findings were statistically significant. These inconsistencies in results highlight the complexity of the relationship between higher-weight social identification and well-being among higher-weight individuals.

**Table 7:** Results for the relationship between weight stigma and various psychological and physical outcomes mediated by higher-weight social identity (For an explanation of statistics presented in this Table, refer to Conclusions in Table 6).

Outcome	Study	Statistical approach	Mediation				Moderated mediation		Summary	*k*^a^	*k* significant %
			a (SE)	b (SE, [95% CI])	Indirect effect (a*b [95% CI])	Direct effect (c’ [95% CI])					
Psychological outcomes (*n* = 5)											
Self-esteem (*n* = 2)										2	100%
	Magallares et al. [30]	Pathway analysis using AMOS	−0.37 (NR)	0.12 (NR)	NR	−0.41 (NR), [NR]	–	–	HWSI was a (partial) mediator between weight stigma and self-esteem. There was a negative relationship between weight discrimination and self-esteem, but a positive relationship between HWSI and self-esteem. However, indirect effects not reported. The model showed an appropriate goodness of fit (*χ*^2^ = 0.14, *p* = 0.92; CFI = 1; NFI = 0.99; RMSE = 0.001		Note: both findings were significant. However, the mediation effect of HWSI was positive in the Magallares et al. [30] study but negative in Wellman et al.’s [45] study.
	Wellman et al. [45]	PB5K	[NR]	−0.16* (0.02)	−0.08* [−0.10 to −0.06]	−0.05* [−0.08 to 0.01]	–	–	HWSI (partially) mediated the negative relationship between weight stigma and self-esteem. Did not report standardised effect size for indirect effect.		
Satisfaction with life (*n* = 1)										1	100%
	Wellman et al. [45]	PB5K	[NR]	−0.08 (0.04)	−0.04 [−0.08 to 0.01]	−0.19* [−0.27 to −0.10]	–	–	HWSI did not mediate the relationship between weight stigma and satisfaction with life.		
Perceived stress (*n* = 1)										1	100%
	Araiza et al. [44]	PB5K	[NR]	0.12** (0.02)	0.04* [CI: 0.02 to 0.06]	0.15* [CI: 0.10 to 0.20]	–	–	HWSI (partially) mediated the negative relationship between weight stigma and perceived stress. Did not report standardised effect size for indirect effect.		
Disordered eating (*n* = 1)										1	100%
Uncontrolled eating	Araiza et al. [44]	PB5K	[NR]	1.56** (0.10)	0.53* [CI: 0.43 to 0.65]	0.38* [CI: 0.16 to 0.61]	–	–	HWSI (partially) mediated the negative relationship between weight stigma and uncontrolled eating. Did not report standardised effect size for indirect effect.		
Emotional eating	Araiza et al. [44]	PB5K	[NR]	0.49** (.05)	0.17* [CI: 0.13 to 0.21]	0.25* [CI: 0.14 to 0.35]	–	–	HWSI (partially) mediated the negative relationship between weight stigma and emotional eating. Did not report standardised effect size for indirect effect.		
Psychological distress (*n* = 1)										1	100%
	Curll and Brown [29]	PB5K	0.25** [0.13, 0.36]	1.04** [0.13, 0.36]	0.26** [0.08, 0.50]	3.12** [2.37, 3.86]	0.15 (0.06), [0.04, 0.29]	Conditional indirect effects: 16th percentile: −0.28 (0.13), [−0.56 to −0.07] 50th percentile: −0.04 (0.08), [−0.20 to 0.11] 84th percentile: 0.14 (0.11), [−0.05 to 0.40]	1. HWSI fully mediated the positive relationship between perceived weight stigma and psychological distress. Medium, significant, positive mediating effect. 2. IWB significantly moderated the relationship between HWSI and psychological distress, *b* = 0.59** 3. IWB significantly moderated the indirect relationship between perceived weight stigma and psychological distress through HWSI. Small, significant positive interaction effect. Low levels of IWB: indirect effect of perceived weight stigma on psychological distress through HWSI was significant and negative. Moderate levels of IWB: indirect effect of perceived weight stigma on psychological distress through HWSI was negative but non-significant. High levels of IWB: indirect effect of perceived weight stigma on psychological distress through HWSI was positive and non-significant.		
Physical (*n* = 1)											
General Health (*n* = 1)										1	100%
	Wellman et al. [45]	PB5K	[NR]	−2.35** (0.25)	−1.10* [−1.41 to 0.82]	−0.93* [−1.45 to 0.41]	–	–	HWSI (partially) mediated the negative relationship between weight stigma and general health. Did not report standardised effect size for indirect effect.		

*NR* not reported, *Italicised* unstandardised, *not italicised* standardised, *PB* PROCESS calculated bootstrapped 95% Cis of X# of samples, *IMM* index of moderated mediation. Acronyms. Predictor: *PWS* perceived weight stigma. Mediator: *HWSI* higher-weight social identity. Moderator: *IWB* internalised weight bias.

*p < 0.05; **p < 0.01.

^a^*k* = number of unique findings reported in the studies.

### Psychological outcomes

There were six unique findings across all studies for psychological outcomes. As shown in Table 7, findings were mixed for self-esteem. Results from Magallares et al.’s [30] path analysis provided support for the RIM model, revealing a negative link between weight stigma and self-esteem but a positive link between higher-weight group identification and this outcome (see links B and C_b_ in Fig. 1). In contrast, Wellman et al. [45] found that weight stigma was associated with lower self-esteem, and higher-weight social identification significantly mediated this relationship. Similarly, Araiza et al. [44] demonstrated that weight stigma was positively associated with higher-weight social identification, which in turn, was linked to increased stress and disordered eating (see links B and C_b_ in Fig. 1). Curll and Brown [29] found a significant, positive indirect effect of weight stigma on psychological distress through higher-weight social identification. However, this indirect effect was found to be moderated by IWB. That is, for participants with low IWB, higher-weight social identification was associated with lower distress; but the opposite was found for those with higher IWB (supporting links B and D in Fig. 1).

### Physical outcomes

As shown in Table 7, higher weight stigma was negatively related to general health and the indirect pathway from weight stigma to general health through group identification was also negative and significant (see links B and C_b_ in Fig. 1).

### Risk of bias assessment

A risk of bias assessment was performed by AH and LB using the CEBMa checklist. All studies were given an overall quality rating of good, with the exception of one (i.e., Wang et al. [46]; see Table 3 in Supplementary Materials), based on the critical appraisal of the risk of potential for selection bias, information bias, measurement or confounding bias. As such, the pattern of findings in this review were not biased by the methodological rigour of the included studies.

## Discussion

This review explored the role of higher-weight social identity as both a risk and protective factor in the effects of weight stigma on physical and psychological well-being. Given the difficulty of combating weight stigma at the societal level [4], understanding these factors is crucial for developing targeted interventions that effectively reduce the harmful effects of weight stigma for higher-weight individuals. This review synthesised 13 studies. Across the 13 studies, seven different measures of weight stigma were used, higher-weight social identity was operationalised in five different ways, and 18 outcome variables were assessed. Thus, the research question was not suitable for a meta-analysis. Ten studies were informed by the WBSIT model and three by the social cure perspective. A general conclusion that can be drawn is that the studies support both the WBSIT and the social cure models, but results were contingent on the outcomes measured and the inclusion of moderators when evaluating the link between higher- weight social identity and well-being.

### Higher-weight social identity as a risk factor

#### Psychological outcomes

Studies informed by WBSIT theory showed mixed results. Major et al. [11] found that higher-weight women experienced greater stress emotions after weight stigmatisation, unlike their lower-weight counterparts. However, another study [47] found no significant impact of higher-weight social identity on this relationship. Moreover, higher-weight social identity, when accompanied by weight stigma, did not exacerbate the negative emotions experienced, such as reduced self-esteem, increased anxiety, post-interaction rumination, and self-consciousness. Similarly, this social identity did not consistently exacerbate the negative impact of weight stigma on executive functioning, as observed by Blodorn et al. [47] and Hunger et al. [13].

Interestingly, two of three findings indicated that higher-weight social identity played an exacerbating role in the link between weight stigma and expectations of weight-based social rejection, while only one finding showed no impact on this outcome. Anticipated rejection has been identified as a mediator between weight stigma and psychological stress, negative emotions and reduced executive performance [13, 47]. Thus, interventions targeting rejection expectations could be implemented to prevent significant downstream psychological issues for higher-weight individuals. For instance, therapies addressing anxiety symptoms may incorporate cognitive bias modification training to address weight-based rejection expectations [48].

Furthermore, in three studies, all findings demonstrated that higher-weight social identity, combined with weight stigma, detrimentally affects outcomes related to consumption behaviours, such as individuals’ belief in their ability to control their diet and their actual tendency to do so. Notably, higher-weight women consumed more high-calorie snacks after weight stigma exposure compared to average-weight individuals [49, 50]. Since self-efficacy is crucial for long-term behavioural self-regulation among higher-weight individuals [51], interventions should prioritise promoting dietary self-efficacy. However, one study found that weight stigma’s negative consequences on alcohol use and disordered eating were worse for lower-weight individuals [37]. Given the prominence of behavioural responses to weight stigmatisation among higher-weight individuals in the WBSIT model, further research assessing these outcomes is necessary, as this study alone is insufficient.

#### Physical outcomes

The current review revealed that all findings from the three studies that assessed physical outcomes indicated that stigma and WBSIT can get “under the skin” to undermine a person’s physical health and their ability to fully participate in life [52]. Specifically, higher-weight social identity was found to significantly increase the harmful effects of weight stigma on physiological stress and partly explained weight stigma’s impact on functional disability among individuals identifying as higher-weight. However, as research is limited, further research in this area is needed.

### Higher-weight social identity as a protective factor

#### Psychological outcomes

Studies informed by the social cure also showed mixed results. One study found that identification protected against the negative impact of weight stigma on self-esteem [30]. In contrast, Wellman et al. [45] reported that higher-weight social identity exacerbated the harmful impact of stigma on self-esteem, and Araiza et al. [44] similarly identified detrimental effects, linking higher-weight social identity to increased stress and unhealthy eating behaviours. Methodological differences may explain these discrepancies. Wellman et al. and Araiza et al. both used large community samples (*n* = 739 and *n* = 1257, respectively), whereas Magallares et al. [30] included 95 participants exclusively from higher-weight associations, possibly influencing their tendency to positively identify with the higher-weight group. Wellman et al. found no evidence that higher-weight social identity exacerbates the negative relationship between weight stigma and life satisfaction. However, this is one study and further research is required.

Importantly, Curll and Brown [29] found that higher perceived weight stigma was associated with stronger identification that, in turn, was associated with higher psychological distress. However, in line with their predictions, this indirect relationship was moderated by IWB. Increased identification was shown to be an effective strategy for coping with the distress associated with stigma, but only when IWB was low. This study provides valuable insights into individual factors that determine when higher-weight social identification can buffer against the harmful effects of weight stigma. It also provides support for strategies (such as, participation in online size acceptance communities) and psychosocial interventions (including group-based treatments) that aim to harness the benefits of a higher-weight social identity.

#### Physical outcomes

One study unexpectedly found that higher-weight social identification exacerbated the harmful effects of weight stigma on general health. However, given the limited research in this area, further studies should examine the influence of individual factors, such as levels of IWB, on these relationships.

#### Limitations of the studies

Most studies in this review that were guided by the WBSIT model assessed higher-weight social identity using either psychological group membership (i.e., self-perceived weight; *n* = 3), or sociological/assigned group membership (i.e., BMI; *n* = 4). Although both forms of identity are important to assess, they are conceptually and operationally different. Whereas psychological group membership reflects self-perception, sociological group membership denotes an assigned membership that may or may not correspond to one’s identity. Psychological membership is more likely to impact outcomes [8] compared to an externally assigned identity that may or may not be internalised. Acknowledging these issues, three studies [13, 37, 53] used self-perceived weight alongside BMI to assess the impact of higher-weight social identity on the harmful effects of weight stigma, all of which found significant moderation effects for both BMI and self-perceived weight. However, it remains uncertain whether mere awareness of group membership is a true measure of social identity, since social identity theory also emphasises the evaluative and affective aspects of identity [19]. Thus, it raises questions about whether a more comprehensive measure of social identity would yield different results.

To comprehensively capture higher-weight social identity, it is necessary to assess both the extent to which group membership contributes to one’s self-concept and to the positive emotional valuation of that membership [14]. Although the former was assessed in the social cure studies through measures of identity centrality, individuals’ positive feelings towards and attachment to (e.g., embracing, rejecting or neutral) the ingroup were not assessed. To better understand the complexity of the higher-weight experience and how to minimise negative outcomes of stigma, research should employ a multidimensional operationalisation of social identity that incorporates affective components, such as satisfaction with and attachment to the ingroup, in addition to identity centrality (see Leach et al. [42]). By doing so, researchers can explore the psychosocial resources available to individuals and determine how they can either facilitate effective coping with stigma or potentially intensify its negative consequences. It is essential to explore the possibility that some individuals may embrace a higher-weight identity, which is likely to result in positive psychological outcomes [54].

Furthermore, the four studies that employed the social cure approach used a correlational design, which restricted the ability to draw causal conclusions. Although the social cure approach is theoretically strong, it is possible that psychological well-being might also influence social identification and weight stigma [29]. Moreover, perceived weight stigma was assessed by reference to participants’ past stigma experiences, and so may have been vulnerable to recall bias [29]. Future research should consider longitudinal methods, such as ecological momentary assessment [55], to measure stigma experiences in real-time, reducing the potential for recall bias.

### Risk of bias

In this review, 13 out of the 14 included studies were of good quality. Therefore, results were not impacted by discrepancies in quality, suggesting there was no bias in the results. However, areas for improvement by researchers include reporting response rates (*n* = 5 studies achieved a satisfactory response rate), and confidence intervals for main results (*n* = 7). None of the studies reported conducting an a priori power analysis. If studies were underpowered this could explain non-significant findings present. Future studies should report adequate information for meaningful conclusions to be drawn.

### Limitations of this review

This review has some limitations. Firstly, whilst revealing important directions for future research, the heterogeneity of findings makes it challenging to conduct meta-analysis and draw definitive conclusions about the role of higher-weight social identity as a risk or protective factor in the relationship between weight stigma and well-being. Secondly, consistent reporting of effect sizes was observed for only three out of the 18 outcomes assessed (i.e., for dietary self-efficacy, cortisol reactivity, and psychological distress), limiting a comprehensive overview of the magnitude of the impact of higher-weight social identity on health outcomes. Thirdly, the scope of this review was limited by the exclusion of unpublished or grey literature, potentially introducing publication bias and excluding relevant information. Future review should include such literature to address these limitations.

## Conclusion

This review aimed to investigate the role of higher-weight social identity as a risk and a protective factor in the relationship between weight stigma and psychological and physical outcomes. Studies that examined higher-weight social identity as a risk factor showed that individuals aware of their higher-weight social categorisation were more likely to expect to be rejected due to their weight, perceive a lack of control over their diet, and engage in increased eating in response to stigma. However, research on other outcomes was limited and inconclusive and prevented more robust conclusions from being drawn. A major limitation of these studies was the lack of assessment of the evaluative and emotional components of higher-weight social identity, opening up new avenues for research. This review also highlighted the strengths of the social cure approach, which revealed that stronger identification with the higher-weight group could mitigate distress and improve self-esteem for certain individuals (e.g., those with low IWB). However, limited research informed by this approach and mixed results impeded definitive conclusions about the buffering role of social identification.

Overall, whilst the impact of a higher-weight social identity requires further investigation, this review suggests promising directions for future research. Researchers should use more comprehensive measures of higher-weight social identity and conduct experimental or longitudinal studies that measure actual weight stigma experiences to validate the predictive power of the social cure approach within the higher-weight group. Additionally, exploring individual factors that influence the social cure effect should be a continued focus of research. In the absence of much needed societal changes to eliminate weight stigma, such research is crucial for the development of effective strategies and psychosocial treatments that can foster a positive social identity and boost the well-being of individuals affected by weight stigma.

### Methodological statement

In compliance with the established guidelines and regulations for conducting systematic reviews, we confirm that all methods utilised in this manuscript were rigorously performed in alignment with the PRISMA (Preferred Reporting Items for Systematic Reviews and Meta-Analyses) guidelines. This includes thorough literature searches, eligibility assessments, data extraction, and quality assessments, all of which adhered to the accepted standards in the field. We are committed to maintaining the integrity and transparency of our research, ensuring that our systematic review contributes meaningfully to the body of knowledge in this area.

## Supplementary information

Higher-Weight Social Identity as a Risk and Protective Factor in the Negative Health Consequences of Weight Stigma: A Systematic Review

## Footnotes

1 Note that in this review, weight stigma refers to “public stigma”, including experienced, anticipated, and perceived stigma (see Emmer et al. [2]) and not to “private stigma” (i.e., internalised weight stigma or self-stigma).
