# Supplementary material for: Higher-weight social identity as a risk and protective factor in the negative health consequences of weight stigma: a systematic review
Source: Int J Obes (Lond). 2025 Apr 16;49(7):1209–28. doi: 10.1038/s41366-025-01755-z (PMC12283344; doi:10.1038/s41366-025-01755-z)
Supplement: Supplementary file 1 — Higher-Weight Social Identity as a Risk and Protective Factor in the Negative Health Consequences of Weight Stigma: A Systematic Review [file 41366_2025_1755_MOESM1_ESM.docx]

**Supplementary Materials**

Table 1

*Six Databases: PsycInfo, CINAHL, Web of Science Core Collection, Medline Complete, Scopus, and Embase*

| Combining search terms for three concepts |  | S10: S3 AND S6 and S9 |  |
| --- | --- | --- | --- |
|  | Concept 1 | Concept 2 | Concept 3 |
|  | Stigma | Higher-Weight | Social Identity |
| Subject heading OR (TI OR AB) | S3: S1 OR S2 | S6: S4 OR S5 | S9: S7 OR S8 |
| TI OR AB | S1: Stigma* OR discrimina* OR bias OR anti-fat OR anti-obes* | S4: weight OR overweight OR obes* OR higher- weight OR fat OR “body size” | S7: “social identi*” OR “fat identi*” OR “group identi*” OR “weight percept*” OR “weight status” OR “self- perce*” OR “self-categor*” OR “higher-weight identi*” |
| PsycINFO thesaurus terms (DE; exact subject heading) | S2: “Discrimination” OR “Stigma” | S5: “Body Size” OR “Obesity” OR  “Overweight” | S8: “Social Identity” OR  “Group Identity” |
| CINAHL (MH; exact subject headings) | “Discrimination” OR  “Perceived  Discrimination” OR “Weight Bias” OR “Stigma” | “Body weight” OR  Obesity | “Social Identity” OR ‘group identity” |
| Medline (MH; exact subject heading) | “Social Discrimination”  OR “weight  discrimination” OR “Social Stigma” OR “Weight Prejudice” | “Overweight” OR “Obesity” OR “Body Weight” OR “Body Size” OR “Weight” | “Social Identification” |
| Web of Science (no thesaurus/subject headings available; used TS) | “Discrimination” OR “Stigma” | “Body Size” OR “Obesity” OR “Overweight” | “Social Identity” OR “Group Identity” |
| EMBASE (/; subject headings) | “Social Stigma” OR “Obesity Stigma” OR “Weight Stigma” OR “Stigma” | Obesity | “Social Identity” |
| Scopus | N/A | N/A | N/A |
|  |  |  |  |

*Note.* Parentheses removed throughout table for ease of reading. S = Search.

# Table 2

*CEBMa Checklist Criteria*

| Criteria Number | Criteria Description |
| --- | --- |
| 1 | Did the study address a clearly focused question/issue? |
| 2 | Is the research method (study design) appropriate for answering the research question? |
| 3 | Is the method section of the participants clearly described? |
| 4 | Could the way the sample was obtained introduce (selection) bias? |
| 5 | Was the sample of participants representative with regard to the population to which  the findings will be referred? |
| 6 | Was the sample size based on pre-study considerations of statistical power? |
| 7 | Was a satisfactory response rate achieved? |
| 8 | Are the measurements (questionnaires) likely to be valid and reliable? |
| 9 | Was the statistical significance assessed? |
| 10 | Are confidence intervals given for the main results? |
| 11 | Could there be confounding factors that haven’t been accounted for? |

Figure 1

*PRISMA Flowchart for the Study Selection Process*


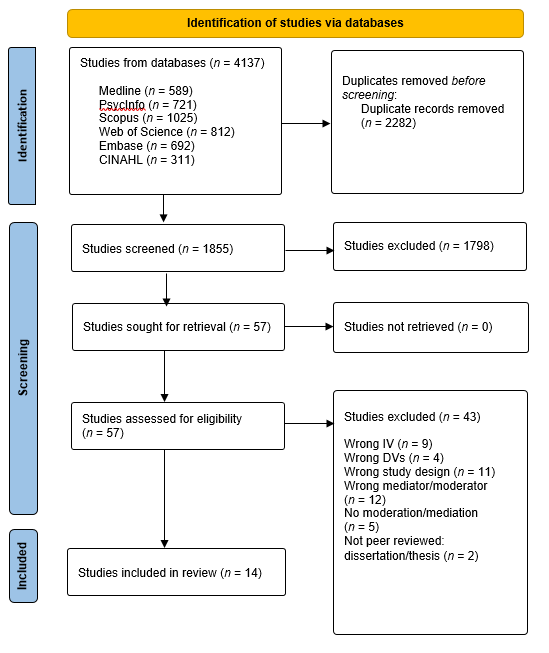


**Table 3**

*Quality Assessment of Included Studies - CEBMa Checklist Results*

| Author(s), Year | Criterion 1 | Criterion 2 | Criterion 3 | Criterion 4 | Criterion 5 | Criterion 6 | Criterion 7 | Criterion 8 | Criterion 9 | Criterion 10 | Criterion 11 |  |  |  |  |  |  |  |  |  |  |  |  |
| --- | --- | --- | --- | --- | --- | --- | --- | --- | --- | --- | --- | --- | --- | --- | --- | --- | --- | --- | --- | --- | --- | --- | --- |
|  | Did the study  address a clearly focused question? | Is the research method  appropriate for answering the research question? | Is the method of selection of the subjects  clearly described? | Could the way the sample was obtained introduce bias? | Was the sample of subjects representative of the population that the finding will  refer to? | Was the sample size based on pre-study consideration of statistical power? | Was a satisfactory response  rate received? | Is the measurement likely to be valid and reliable? | Was the statistical significance assessed? | Are confidence intervals given for the main  results? | Could there be  confounding factors that haven’t been accounted for? | Quality Rating  (Good, Fair, or Poor) |  |  |  |  |  |  |  |  |  |  |  |
| Araiza et al. (2025)  Blodorn et al. (2016) | Yes    Yes | Yes  Yes | Yes  Yes | Yes  No | Yes  No | No  No | Can’t say  N/A | Yes  Yes | Yes  Yes | Yes  No | Yes  Yes | Good  Good |  |  |  |  |  |  |  |  |  |  |  |
| Brochu and Dovidio  (2013) | Yes | Yes | Yes | No | No | No | N/A | Yes | Yes | No | Yes | Good |  |  |  |  |  |  |  |  |  |  |  |
| Curll and Brown  (2020) | Yes | Yes | Yes | Yes | Yes | No | Can’t say | Yes | Yes | Yes | Yes | Good |  |  |  |  |  |  |  |  |  |  |  |
| Himmelstein et al. (2015) | Yes | Yes | Yes | No | No | No | N/A | Yes | Yes | Yes | No | Good |  |  |  |  |  |  |  |  |  |  |  |
| Hunger et al. (2018) | Yes | Yes | Yes | No | No | No | N/A | Yes | Yes | Yes | No | Good |  |  |  |  |  |  |  |  |  |  |  |
| Lee et al. (2021) | Yes | Yes | Yes | Yes | No | No | Can’t say | Yes | Yes | Yes | No | Good |  |  |  |  |  |  |  |  |  |  |  |
| Schafer and Ferraro (2011) | Yes | Yes | Yes | Yes | Yes | No | Yes | Yes | Yes | No | No | Good |  |  |  |  |  |  |  |  |  |  |  |
| Magallares et al. (2014) | Yes | Yes | Yes | Yes | No | No | Can’t say | Yes | Yes | No | Yes | Good |  |  |  |  |  |  |  |  |  |  |  |
| Major et al. (2012) | Yes | Yes | Yes | No | No | No | Yes | Yes | Yes | No | No | Good |  |  |  |  |  |  |  |  |  |  |  |
| Major et al. (2014) | Yes | Yes | Yes | No | No | No | Yes | Yes | Yes | No | No | Good |  |  |  |  |  |  |  |  |  |  |  |
| McCleary-Gaddy et al. (2018) | Yes | Yes | Yes | Yes | No | No | Can’t say | Yes | Yes | No | No | Good |  |  |  |  |  |  |  |  |  |  |  |
| Wang et al. (2020) | Yes | Yes | Yes | Yes | No | No | Yes | Yes | No | No | Yes | Fair |  |  |  |  |  |  |  |  |  |  |  |
| Wellman et al. (2022) | Yes | Yes | Yes | Yes | Yes | No | Can’t say | Yes | Yes | Yes | No | Good |  |  |  |  |  |  |  |  |  |  |  |

**Appendix A**

**Reviewed Articles**

Araiza, A. M., Vieira Zaidan, A. C., Wijayatunga, N. N., & Wellman, J. D. (2025). Weight discrimination as a predictor of stress and eating: The role of identifying as “fat.” *Appetite*, *206*(01), 107772-. https://doi.org/10.1016/j.appet.2024.107772

Blodorn, A., Major, B., Hunger, J., & Miller, C. (2016). Unpacking the psychological weight of weight stigma: A rejection-expectation pathway. *Journal of Experimental Social Psychology, 63*(1), 69-76. https://doi.org/10.1016/j.jesp.2015.12.003

Brochu, P. M., & Dovidio, J. F. (2013). Would you like fries (380 calories) with that? Menu labeling mitigates the impact of weight-based stereotype threat on food choice. *Social Psychological and Personality Science, 5*(4), 414-421. https://doi.org/10.1177/1948550613499941

Curll, S. L., & Brown, P. M. (2020). Weight stigma and psychological distress: A moderated mediation model of social identification and internalised bias. *Body Image, 35*(1), 207-216. https://doi.org/https://dx.doi.org/10.1016/j.bodyim.2020.09.006

Himmelstein, M. S., Incollingo Belsky, A. C., & Tomiyama, A. J. (2015). The weight of stigma: Cortisol reactivity to manipulated weight stigma. *Obesity, 23*(2), 368-374.

Hunger, J. M., Blodorn, A., Miller, C. T., & Major, B. (2018). The psychological and physiological effects of interacting with an anti-fat peer. *Body Image, 27*(1), 148-155. https://doi.org/10.1016/j.bodyim.2018.09.002

Lee, K. M., Hunger, J. M., & Tomiyama, A. J. (2021). Weight stigma and health behaviors: Evidence from the eating in America study. *International Journal of Obesity, 45*(7), 1499-1509. https://doi.org/10.1038/s41366-021-00814-5

Magallares, A., Morales, J. F., & Rubio, M. Á. (2014). Group identification, discrimination and psychological health in an obese sample. *International Journal of Psychology and Psychological Therapy, 14*(3), 421-431. https://search.ebscohost.com/login.aspx?direct=true&AuthType=shib&db=psyh&AN=2014- 47636-008&site=ehost-live&scope=site&custid=s5501413

Major, B., Eliezer, D., & Rieck, H. (2012). The psychological weight of weight stigma*. Social Psychological and Personality Science, 3*(6), 651-658. https://doi.org/10.1177/1948550611434400

Major, B., Hunger, J. M., Bunyan, D. P., & Miller, C. T. (2014). The ironic effects of weight stigma. *Journal of Experimental Social Psychology, 51*(1), 74-80. https://doi.org/10.1016/j.jesp.2013.11.009

McCleary-Gaddy, A. T., Miller, C. T., Grover, K. W., Hodge, J. J., & Major, B. (2019). Weight stigma and hypothalamic–pituitary–adrenocortical axis reactivity in individuals who are overweight. *Annals of Behavioral Medicine, 53*(4), 392-398. https://doi.org/10.1093/abm/kay042

Schafer, M. H., & Ferraro, K. F. (2011). The stigma of obesity: Does perceived weight discrimination affect identity and physical health? *Social Psychology Quarterly, 74*(1), 76–97. https://doi.org/10.1177/0190272511398197

Wang, Z., Wang, B., Hu, Y., Cheng, L., Zhang, S., Chen, Y., & Li, R. (2020). Relationships among weight stigma, eating behaviors and stress in adolescents in Wuhan, China. *Global Health Research and Policy, 5*(1), 8-18. https://doi.org/https://dx.doi.org/10.1186/s41256-020-00138-3

Wellman, J. D., Araiza, A. M., Nguyen, T. V. C., Beam, A. J., & Pal, S. (2022). Identifying as fat: Examining weight discrimination and the rejection-identification model. *Body Image, 41*(1), 46-51. https://doi.org/https://dx.doi.org/10.1016/j.bodyim.2022.02.008
